# Rapidly Progressive Dementia with Asymmetric Rigidity Following ChAdOx1 nCoV-19 Vaccination

**DOI:** 10.14336/AD.2021.1102

**Published:** 2022-06-01

**Authors:** Sankha Shubhra Chakrabarti, Ashutosh Tiwari, Sumit Jaiswal, Upinder Kaur, Ishan Kumar, Amit Mittal, Anup Singh, Kunlin Jin, Sasanka Chakrabarti

**Affiliations:** ^1^Department of Geriatric Medicine, Institute of Medical Sciences, Banaras Hindu University, Varanasi, UP, India.; ^2^Department of Neurology, All India Institute of Medical Sciences, Rishikesh, Uttarakhand, India.; ^3^Department of Geriatric Medicine, Institute of Medical Sciences, Banaras Hindu University, Varanasi, UP, India.; ^4^Department of Pharmacology, Institute of Medical Sciences, Banaras Hindu University, Varanasi, UP, India.; ^5^Department of Radiodiagnosis and Imaging, Institute of Medical Sciences, Banaras Hindu University, Varanasi, UP, India.; ^6^Department of Radiology, Maharishi Markandeshwar (deemed to be) University, Mullana, Haryana, India.; ^7^Department of Geriatric Medicine, Institute of Medical Sciences, Banaras Hindu University, Varanasi, UP, India.; ^8^Department of Pharmacology and Neuroscience, University of North Texas Health Science Center, Fort Worth, Texas, USA.; ^9^Department of Biochemistry and Central Research Cell, Maharishi Markandeshwar (deemed to be) University, Mullana, Haryana, India

**Dear Editor,**

**Figure 1.**
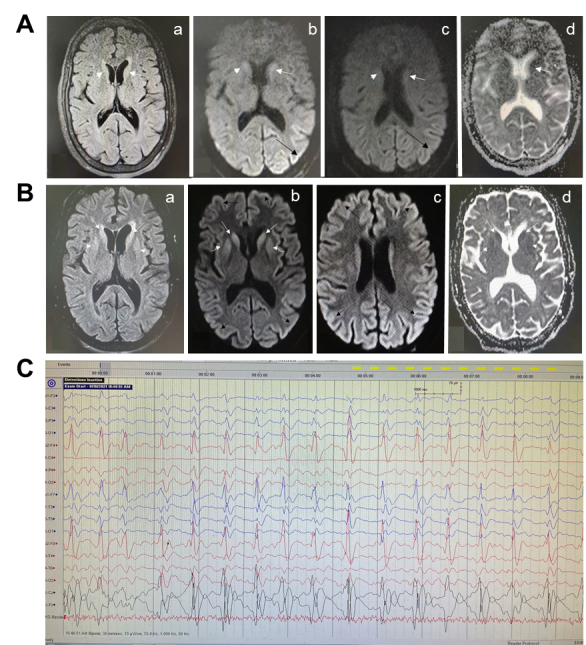
Specialized neurological investigations in the patient. (A) Magnetic resonance imaging of the brain performed after admission. Axial FLAIR (a), Diffusion b1000 (b, c) and ADC map (d) images show FLAIR hyperintensity in bilateral caudate head which shows diffusion restriction (white arrows). Further, there is patchy diffusion restriction in left sided gyri in posterior parietal and occipital lobes. (B) Magnetic resonance imaging of the brain performed after 16 days of initial imaging. Axial FLAIR (a), Diffusion b1000 (b, c) and ADC map (d) images of the same patient 16 days later shows increased degree of FLAIR hyperintensity and diffusion restriction in bilateral caudate (white arrow) as well as involvement of bilateral putamina (curved arrow). There is diffuse diffusion restriction in cerebral cortex in bilateral cerebral hemispheres (black arrow). Cortical ribboning is visible in diffusion weighted images. (C) Electroencephalogram of the patient. Electroencephalogram showing bilateral biphasic and triphasic sharp wave periodic discharges of around one Hz, with slowing of background activity.

A previously healthy woman in her 60s developed social isolation and behavioural abnormality in the form of moving around un-purposefully, irrelevant verbal responses and refusal to take food, one day after receiving the second dose of ChAdOx1 nCoV-19 vaccination (COVISHIELD). Her first dose (dose interval 84 days) was followed by an uneventful course and one day of mild fever. Her current condition progressed rapidly with onset of confusion, forgetfulness, and hallucinations within the next 5 days. Over the next two days, the patient developed difficulty in walking and echolalia. She was prescribed amantadine, trihexyphenidyl and clonazepam by a local practitioner but she showed further deterioration in the form of irritability, incoherent speech, aggravated forgetfulness, auditory and visual hallucinations, abnormal movements of limbs, and jaw and neck dystonia. The drugs were withdrawn, and the patient was shifted to quetiapine without any significant improvement in her condition. At presentation to the Geriatric services, she was in delirium and was immobile. She was afebrile with stable vitals and a Glasgow Coma Score of 9 (eyes 3, verbal 2, motor 4). Bilateral plantars were upgoing and she had asymmetric rigidity, more on the left side. Her routine haematological and biochemical labs including creatine phosphokinase levels and thyroid profile were within normal limits. Cerebrospinal fluid analysis was unremarkable including a panel for common regional etiologies of viral encephalitis. Serum panel for autoimmune encephalitis was also negative for common antibodies including anti-LGI1. The patient had a serum anti-Spike protein IgG titre of 1891.5 AU/mL (non-reactive<50 AU/mL) at 37 days post-second dose. An initial magnetic resonance imaging (MRI) of the brain had revealed FLAIR hyperintensity in bilateral caudate heads which showed diffusion restriction and also patchy diffusion restriction in the left posterior parietal and occipital gyri (Fig. 1A). In the absence of clinical improvement, a repeat imaging was performed after 16 days (Fig. 1B) which revealed an increase in the degree of FLAIR hyperintensity and diffusion restriction in bilateral caudate nuclei as well as involvement of bilateral putamina, with diffuse diffusion restriction in the cerebral cortex in bilateral hemispheres. A cortical ribboning pattern of hyperintensity was observed in the diffusion image. An electroencephalogram (EEG) revealed bilateral biphasic and triphasic sharp wave periodic discharges of around one Hz, with slowing of background activity (Fig. 1C). The patient was managed conservatively with a trial of dexamethasone, empirical broad-spectrum antibiotics, and antiepileptics but had no significant improvement. Later, bromocriptine was added which resulted in some improvement in her limb rigidity. However, overall neurologic deterioration continued over the next two weeks, and she developed respiratory distress and shock to which she finally succumbed after one month of admission. The possibility of a secondary infection leading to sepsis and shock, and eventually resulting in fatality exists. Although radiological and EEG findings in our patient are suggestive of a prion disease like pathology, we could not perform measurement of 14-3-3 protein in cerebrospinal fluid or post-mortem studies of the brain to confirm the diagnosis in this patient.

Prion-like neurodegenerative conditions following COVID-19 vaccination have not been reported, though there are a couple of reports where COVID-19 has been followed by prion disease with characteristic EEG and MRI findings [1, 2]. The authors in these cases did not provide any definitive mechanistic link between COVID-19 and prion-like neurodegeneration. However, it was suggested that the hyperinflammatory state with high levels of pro-inflammatory cytokines commonly associated with COVID-19 might have triggered central nervous system-neuroinflammation through activation of astrocytes and microglia which could have facilitated a prion-like pathology [2]. Such a mechanism is plausible because a neuroinflammation element in the pathogenesis of prion disease is corroborated both in human Creutzfeldt-Jakob disease and experimental animal models of prion disease [3]. Interestingly, some recent studies using the aggregation-prediction server- AGGRESCAN and protein-protein docking techniques have indicated that the S1 subunit of the spike-protein of SARS-CoV-2 can interact with other aggregation-prone proteins of the brain like amyloid beta, prion protein, α-synuclein, and tau, presumably through heparin-binding domains, to form homo or hetero-polymers resembling amyloid fibrils which may play a pivotal role in the neurodegenerative process of the misfolded protein disorders [4, 5]. The molecular docking studies further suggest that the interaction of the S-protein with prion protein is stronger than with amyloid beta, tau or α-synuclein [5]. In addition, like other prion proteins, the S-protein also contains several prionogenic domains (PrDs) [6]. Thus, a direct toxic action of the S-protein, triggering a neurodegenerative condition mimicking a prion disease-like pathology is also a possibility.

In the context of the present case in which rapidly progressive dementia with atypical brain MRI and EEG changes developed following COVID-19 vaccination, two possibilities may be considered, a direct toxicity of the S protein or the toxicity of anti-S protein antibodies. The adenovirus vector or lipid nanoparticle (LNP) encapsulated mRNA encoding full-length S protein (or various modifications of it) could enter different types of host cells at the site of injection.The host’s synthetic machinery would then lead to the expression of the S protein which could be presented on the cell surface or secreted outside [7-9]. When the S protein or any derivative of it is presented by the antigen-presenting cells (APCs) to immune cells at the draining lymph nodes, a robust adaptive immune response by different types of activated B and T lymphocytes is elicited [7-9]. Although the expression of the protein may be limited primarily to the cells including APCs of the local site of injection of vaccine and draining lymph nodes, some degree of expression may also occur in distant organs because of systemic spread, as has been shown for some mRNA vaccines [10, 11]. The bio-distribution and kinetics of S protein expression after COVID-19 vaccination are not known.These parameters were not examined in multiple animal studies which were conducted to assess the immune response and protective actions of various COVID-19 vaccines [12, 13]. However, it is plausible that in occasional individuals, following COVID-19 vaccination, an increased or prolonged expression of S protein may take place leading to direct toxicity. Thus, in the present case, interaction of S protein with aggregation-prone proteins of the brain could be the trigger for neurodegeneration. The alternative possibility could involve anti-S protein antibodies cross-reacting with neural tissue antigens of the host producing a kind of auto-immune response. Such a mechanism mediated by cross-reactive antibodies might have been responsible in the current case similar to many reported complications following COVID-19 vaccines, such as Guillain-Barre syndrome, immune thrombotic thrombocytopenia and stroke events, and exacerbations of demyelinating diseases [14-16]. It is important to note that a recent *in vitro* study identified multiple host antigens from diverse tissues including the brain that could cross-react to varying extent with antibodies specific to SARS-CoV-2 proteins including the Spike [17]. The short interval between vaccine dose and development of symptoms, and the lack of elevation in cerebrospinal fluid protein levels, makes direct toxicity rather than auto-antibody generation a more likely possibility. In the absence of more detailed studies, both possibilities are however conjectural. Finally, though a coincidental occurrence of the dementia syndrome in this patient cannot be ruled out, the temporal association is in favour of a post-vaccination complication. It is also possible that a latent prion pathology was unmasked by the immunological changes following vaccination.

Recently we have demonstrated favourable short-term safety of ChAdOx1 based COVID-19 vaccine (COVISHIELD) in a real-world prospective observational study [18]. Although neurological adverse events following COVID-19 vaccination such as in the current case may be rare, they merit research into causality and enhance the need for vigilant long-term post-marketing surveillance.
